# Effect of propofol and ciprofol on the euphoric reaction in patients with painless gastroscopy: A prospective randomized controlled trial

**DOI:** 10.1016/j.heliyon.2024.e30378

**Published:** 2024-04-25

**Authors:** Teng Li, Jin Zhang, Zhouliang Liu, Yao Lu, Chuhao Gong, Dan Han, Ying Wu, Kailun Gao, Lei Heng, Liwei Wang, Peng Peng

**Affiliations:** a XuZhou Clinical School of Xuzhou Medical University, Xuzhou, China; b Department of Anesthesiology, Xuzhou Renci Hospital, Xuzhou, China; c Department of Anesthesiology, Xuzhou Central Hospital, Xuzhou, China; d Department of Anesthesiology, Xuzhou Cancer Hospital, Xuzhou, China; e Department of Anesthesiology, Xuzhou New Healthy Geriatric Hospital, Xuzhou, China; f Department of Anesthesiology, the Affiliated Xuzhou Hospital of JiangSu University, Xuzhou, China

**Keywords:** Ciprofol, Propofol, Euphoria, Addiction, Gastroscope

## Abstract

**Objective:**

To explore the effects of propofol and ciprofol on patient euphoric reactions during sedation in patients undergoing gastroscopy and to investigate potential factors that may influence euphoric reactions in patients.

**Methods:**

A total of 217 patients were randomly divided into two groups: the propofol group (P group, n = 109) and the ciprofol group (C group, n = 108). The patients in the P group were given 2 mg/kg propofol, and those in the C group were given 0.5 mg/kg ciprofol. The patients were assessed using the Addiction Research Center Inventory-Chinese Version (ARCI-CV) to measure euphoric reactions at three time points: preexamination, 30 min after awakening, and 1 week after examination. Anxiety, depression, and sleep status were evaluated using appropriate scales at admission and 1 week after the examination. The dream rate, sedative effects, vital sign dynamics, and adverse reactions were documented during the sedation process.

**Results:**

After 30 min of awakening, the P group and C group showed no statistically significant differences in the mean morphine-benzedrine group (MBG) score (8.84 vs. 9.09, *P* > 0.05), dream rate (42.2 % vs. 40.7 %, *P* > 0.05), or MBG score one week after the examination (7.04 vs. 7.05, *P* > 0.05). The regression analysis revealed that sex, dream status, Alcohol Use Disorders Identification Test (AUDIT) score, and examination time had notable impacts on the MBG-30 min score. No statistically significant differences were observed in sedative effects, anxiety, depression, or sleep status between the two groups (*P* > 0.05). The incidence of injection pain and severe hypotension was significantly lower in the C group (*P* < 0.05), and hemodynamics and SpO_2_ were more stable during sedation (*P* < 0.05).

**Conclusion:**

There was no significant difference between propofol and ciprofol in terms of euphoria experienced by patients after sedation in patients undergoing gastroscopy. Ciprofol has demonstrated addictive potential similar to that of propofol, warranting careful attention to its addictive potential during clinical application.

## Highlights

• 1. Study comparing ciprofol to propofol for post-gastroscopy sedation euphoria reaction for the first time
• There was no difference in the incidence of euphoric reactions among patients receiving ciprofol or propofol.
• This study provides a new perspective for clinical anesthesia selection and addiction-related research.
• The results of this study could provide useful guidance for clinical practice and further research.

## 1 Introduction

Due to its rapid onset of action and short half-life, propofol is increasingly used in the fields of intravenous general anesthesia and ICU sedation [1]. In recent years, it has also gradually become used for sedation in patients undergoing gastroscopy and is one of the most commonly used drugs for sedation [[2], [3], [4]]. However, the increasing use of propofol has led to the emergence of adverse reactions, including respiratory and circulatory depression as well as injection pain, in clinical practice [[5], [6], [7], [8], [9]]. Remarkably, a significant number of patients administered propofol may experience euphoric reactions [[10], [11], [12]], which raises concerns regarding potential abuse and addiction [10,13]. More worrisome is that even a single dose of propofol can lead to addiction [13,14]. Moreover, even among healthcare workers who are knowledgeable about the pharmacokinetics and pharmacodynamics of the drug, the mortality associated with propofol misuse may be as high as 33 % [15]. We cannot ignore the high mortality rate of people with drug addiction. Consequently, some scholars have proposed including propofol in the list of controlled substances for psychotropic substances [16]. This emphasizes the necessity of studying the euphoric reaction and addictive characteristics of sedatives during sedation in patients undergoing gastroscopy.

Ciprofol is a short-acting intravenous sedative that is structurally modified from propofol and exhibits improved efficacy [17]. Compared with propofol in gastroenteroscopy, ciprofol has a lower incidence of respiratory depression, less influence on hemodynamics in elderly patients, and a lower incidence of injection pain [18,19]. Under general anesthesia, the adverse events (including intubation reactions) of ciprofol were significantly lower than those of propofol, and the effects of ciprofol on the circulatory system were slightly less pronounced [18]. Ciprofol has promising clinical applications and has gradually been used in surgical anesthesia and sedation [[20], [21], [22]]. However, the occurrence of euphoric reactions in patients after anesthesia with ciprofol has not been studied, and further exploration is needed to determine whether the occurrence of euphoric reactions is lower in patients sedated or anesthetized with this drug. The present study aimed to alleviate the social burden of propofol addiction and provide effective alternative treatments.

Understanding the euphoric reaction to the use of these sedatives in patients undergoing gastroscopy is essential for guiding clinical practice and ensuring patient safety. Therefore, this study aimed to prospectively evaluate and compare the effects of propofol and ciprofol on the euphoric reaction to sedation in patients undergoing gastroscopy.

## 2 Methods

### 2.1 Trial design and patients

This was a prospective, randomized, double-blind, controlled trial conducted at Xuzhou Renci Hospital. The research protocol received approval from the Ethics Committee of Xuzhou Renci Hospital (XZRCLL-KT-202307001) and adhered strictly to the ethical principles outlined in the Declaration of Helsinki. Additionally, the study was registered at chictr.org.cn (ChiCTR2300076048), and informed consent was obtained from all participants prior to their inclusion. The participants were patients who underwent sedation during gastroscopy at Xuzhou Renci Hospital between September and November. 2023. The inclusion criteria were as follows: (1) patients aged 18–65 years who underwent sedation during gastroscopy and completed the examination successfully; (2) patients with ASA grades I-II; and (3) patients with a body mass index of 18–30 kg/m^2^. The exclusion criteria were as follows: (1) patients with contraindications to general anesthesia or a history of sedation/anesthesia accidents; (2) patients with allergies to eggs, beans, propofol or ciprofol; (3) patients who were lactating or pregnant; (4) patients with a history of drug abuse, major physical or mental trauma, or neuropsychiatric disease. Psychological assessments were conducted under the guidance of a qualified psychiatrist.

### 2.2 Anesthesia method and assessments

The randomization number table was computer-generated by a nurse. Patients who met the criteria were given propofol (2 mg/kg) or ciprofol (0.5 mg/kg) according to random numbers. Nurses distributed prenumbered syringes containing propofol or ciprofol (with the same physical appearance) based on random numbers. Patients, endoscopists, follow-up personnel and statisticians, but not nurses, were unaware of the grouping situation. The preparation for gastroscopy was carried out in accordance with national guidelines [23]. Venous access was established immediately after the patient entered the examination room, and the relevant vital signs were monitored. Oxygen was provided via a nasal oxygen tube at a flow rate of 5 L/min until the patients completed gastroscopy and were fully awake. Patients in the P group and C group were given 2 mg/kg propofol and 0.5 mg/kg ciprofol, respectively, and the infusion time was 30 s ± 5 s. If the MOAA/S score was still >1 after 2 min, propofol or ciprofol was needed (for which the supplementary dose was 1/4 of the initial loading dose and the supplementary interval was not less than 2 min). A gastroscope was inserted when the MOAA/S score was ≤1, and fluctuations in vital signs or body movements during the procedure were recorded. During the maintenance phase, supplementary doses were given for signs of agitation or insufficient sedation, and the procedure was repeated every 2 min as needed. Dopamine (1–2 mg) was administered intravenously when the blood pressure decreased by more than 30 %. Atropine (0.5 mg) was administered intravenously if the heart rate was <60 beats/min. When the SpO_2_ was <90 % for 10 s, a jaw push was used to open the airway. After the examination, the patients were sent to the recovery room for observation. When they regained consciousness, postoperative recovery was evaluated using the modified Aldrete score every 1 min, and the readiness for hospital discharge score was ≥9. Demographic information, including sex, height, weight, body mass index (BMI), alcohol consumption (AUDIT), smoking status, underlying diseases, anxiety (self-rating anxiety scale, SAS), depression (Patient Health Questionnaire, PHQ-9), sleep quality (The Pittsburgh Sleep Quality Index, PSQI), and MBG(Morphine–Benzedrine Group), was collected before examination. A multiparameter monitor was used to continuously monitor systolic blood pressure (SBP), diastolic blood pressure (DBP), mean arterial pressure (MAP), heart rate (HR), finger pulse oxygen saturation (SpO_2_), and electrocardiogram. Adverse drug reactions, adverse events, and serious adverse events were recorded during the examination, the MBG score (MBG-30 min) was recorded 30 min after the patients were awake, the patients were asked about their dreams, and the patients were followed up by questionnaire or telephone one week after the examination.

## 3 Primary outcome

The primary outcome was the patient's MBG score after awakening for 30 min (MBG-30 min).

## 4 Secondary outcomes

Secondary outcome measures: (1) Incidence and content of dreams during sedation. (2) MBG score (MBG-1w), anxiety score (SAS-1w), depression score (PHQ-9-1w) and sleep status score (PSQI-1w) at 1 week after gastroscopy.

### 4.1 Sedative effect

(1. The induction time (from the beginning of drug administration to a modified vigilance/sedation score (MOAA/S) ≤ 1), awake time (the time of MOAA/S score = 5 points for 3 consecutive times after the examination), and recovery time (the time of opening eyes to an Aldrete score ≥9 points) were recorded. Endoscopists and anesthesiologists were asked about their satisfaction at the end of gastroscopy (0-10), and higher scores indicate greater satisfaction.
(2. SBP, DBP, MAP, HR and SpO_2_ were measured at admission (T0), after the first administration (T1) and at the end of the examination (T2). Vital signs were collected every 5 min during the examination.
(3. The incidence of adverse reactions during sedation (choking cough, injection pain, involuntary movement, nausea and vomiting, dizziness, and low blood oxygen saturation (SpO_2_ ≤ 95 %) and severe low blood oxygen saturation (SpO_2_ ≤ 90 %) (if SpO_2_ ≤ 90 % and duration is more than 10 s, the anesthesiologists should provide jaw support, increase oxygen concentration, open airway and assist ventilation if necessary); bradycardia; hypotension, MAP ≤65 mmHg or MAP ≤20 % reduction from baseline; and severe hypotension (MAP ≤30 % reduction from baseline).

### 4.2 Questionnaires

The Addiction Research Center Inventory (ARCI) scale was developed by the National Institute of Mental Health Addiction Research Center (United States). The Morphine-Benzedrine Group (MBG) is a subscale of the addiction research center inventory (ARCI), which measures drug euphoric effects and serves as a diagnostic tool to assess whether patients feel euphoric. The MBG included items 1 to 16, and the sum of the individual items was the euphoric effect factor score. The Chinese version of the MBG used in this study was translated by experts, and its reliability and validity were tested [10]. The Self-rating Anxiety Scale (SAS) is an internationally accepted tool for self-assessment of anxiety symptoms. It is simple, time-saving, easy to master, and can quickly reflect the subjective feeling of anxiety of the subjects. Each item was scored 4 points, for a total possible score of 100 points. The higher the total score is, the greater the anxiety level [24]. The Patient Health Questionnaire (PHQ-9) can be used to evaluate depression in patients, and the higher the score of the 9 indicators on the PHQ-9 is, the more severe the depression is [25]. The Pittsburgh Sleep Quality Index (PSQI) score, which has 32 items, was used to evaluate the sleep quality of patients. A high PSQI total score usually indicates poor sleep quality [26].

### 4.3 Statistical analysis

According to previous studies, the mean ± standard deviation of the MBG at 30 min was 8.67 ± 4.6 in patients receiving propofol sedation during gastroscopy [10]. Based on 80 % efficacy, the bilateral significance level was 0.05, and a 2-point change in the patient's MBG score after examination was considered clinically important. The needed sample size was 85 subjects per group. Based on a potential dropout rate of 20 %, 107 patients were included in each group. Assuming that the differences are subjectively selected by psychiatrists and anesthesiologists, both intention-to-treat analysis and per-protocol analysis were performed on outcome indicators to ensure consistent conclusions.

All the statistical analyses and figures were generated using SPSS 26.0 and GraphPad Prism 9.0, and the normality of the data distribution was determined using the Kolmogorov‒Smirnov test for quantitative data. Quantitative data that conformed to a normal distribution are expressed herein as the mean ± standard deviation (‾x ± s), and intergroup comparisons were conducted using two independent sample t tests. Nonnormally distributed econometric data are represented by medians (Ms) and interquartile intervals (IQRs); intergroup comparisons were performed using the Mann‒Whitney *U* test, and repeated measures at different time points between groups were analyzed using repeated measures analysis of variance. Count data are expressed as rates and were analyzed using the χ^2^ test or Fisher's exact probability method. The rank sum test was used for comparisons of hierarchical data. Multiple regression analysis were performed on possible factors such as age, sex, education level, BMI, ASA grade, smoking status, AUDIT score, anxiety status, depression status, dream rate, sedation time, awake time, and recovery time. *P* < 0.05 indicated a statistically significant difference.

## 5 Results

### 5.1 Baseline demographics and clinical characteristics

**Fig. 1:**
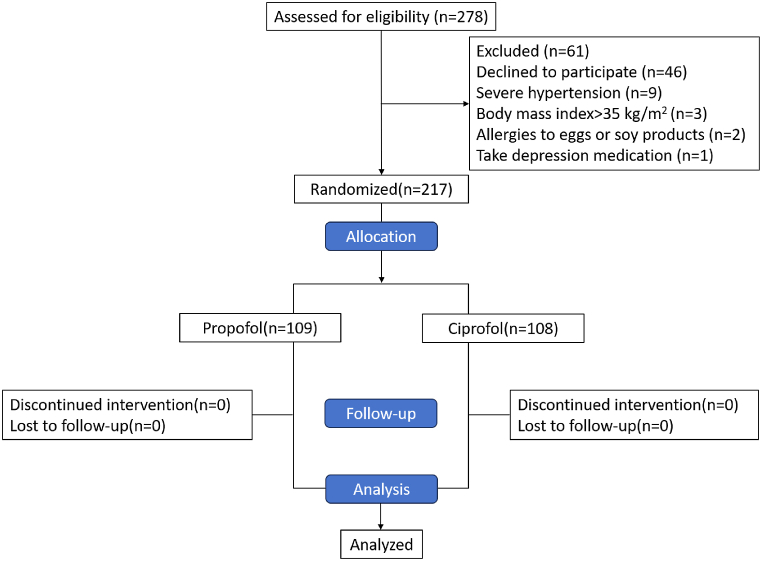
Flowchart of patient enrolment in the study.

A total of 278 patients were evaluated; 6 l did not meet the exclusion criteria, and the exclusion criteria were as follows: refusal to provide informed consent (n = 46), severe hypertension (n = 9), body mass index>30 kg/m^2^ (n = 3), allergy to eggs or soy products (n = 2), or use of antidepressants (n = 1). Finally, 217 patients underwent randomization and completed the examinations(Fig. 1). There was no significant difference in demographic characteristics between the two groups of patients (*P* > 0.05, Table 1). The baseline data included demographic and preexamination emotional and sleep status, as detailed in Table 1.

**Table 1:** Demographic characteristics of the two groups.

	P group (n = 109)	C group (n = 108)	t/χ^2^/Z	*P* value
Age (yr)	47.34 ± 11.20	46.36 ± 12.33	0.612	0.541
Sex, n(%)			0.006	0.940
Male	49(45.0 %)	48(50.0 %)		
Female	60(55.0 %)	60(50.0 %)		
Height (cm)	166.61 ± 9.14	166.65 ± 6.99	−0.039	0.969
Weight (kg)	65.10 ± 9.91	64.39 ± 8.97	0.555	0.580
BMI (kg/m^2^)	23.42 ± 2.79	23.19 ± 3.01	0.589	0.556
Education			0.580	0.748
Junior high school or below	16(14.7)	20(18.5)		
High school and junior College	32(29,4)	30(27.8)		
University or above	61(56)	58(53.7)		
ASA n (%)			0.002	0.960
Ⅰ	71(65.1)	70(64.8)		
Ⅱ	38(34.9)	38(35.2)		
Improved Mallampati score n(%)			0.840	0.657
Ⅰ	54(49.5)	54(50)		
Ⅱ	32(29.4)	36(33.3)		
Ⅲ	23(21.1)	18(16.7)		
Comorbidity, n (%)				
Hypertension, n (%)	41(37.6)	44(40.7)	0.222	0.637
Diabetes, n (%)	23(21.1)	19(17.6)	0.428	0.513
Cardiovascular and cerebrovascular diseases, n (%)	10(9.2)	4(3.7)	2.690	0.101
History of smoking, n (%)	26(23.9)	22(20.4)	0.382	0.537
AUDIT	4(3,5.5)	4(2.25,5.75)	−0.388	0.698
High risk≥8 n (%)	13(11.9)	12(11.1)	0.035	0.851
Dream frequency (week)			3.059	0.383
Never	43(39.4)	48(44.4)		
Less than once per week	46(42.2)	45(41.7)		
Several times per week	13(11.9)	13(12)		
Almost every day	7(6.4)	2(1.9)		
SAS	45.64 ± 7.13	45.06 ± 6.34	0.640	0.523
PHQ-9	3.20 ± 2.05	3.56 ± 2.24	−1.246	0.214
PSQI	8.90 ± 2.06	8.81 ± 2.02	0.305	0.761
MBG	3.84 ± 1.45	3.77 ± 1.32	0.490	0.689

BMI, body mass index. ASA, American Society of Anesthesiology. SAS, self-rating anxiety scale. PHQ-9, Patient Health Questionnaire. AUDIT, alcohol use disorders identification test. PSQI, Pittsburgh Sleep Quality Index. The MGB is a morphine-amphetamine subscale used to assess euphoria. The PCAG, pentobarbital chlorpromazine ethanol subscale was used to assess residual sedation. The values are presented as the means ± SDs or numbers (percentages).

### 5.2 Primary outcome

**Fig. 2:**
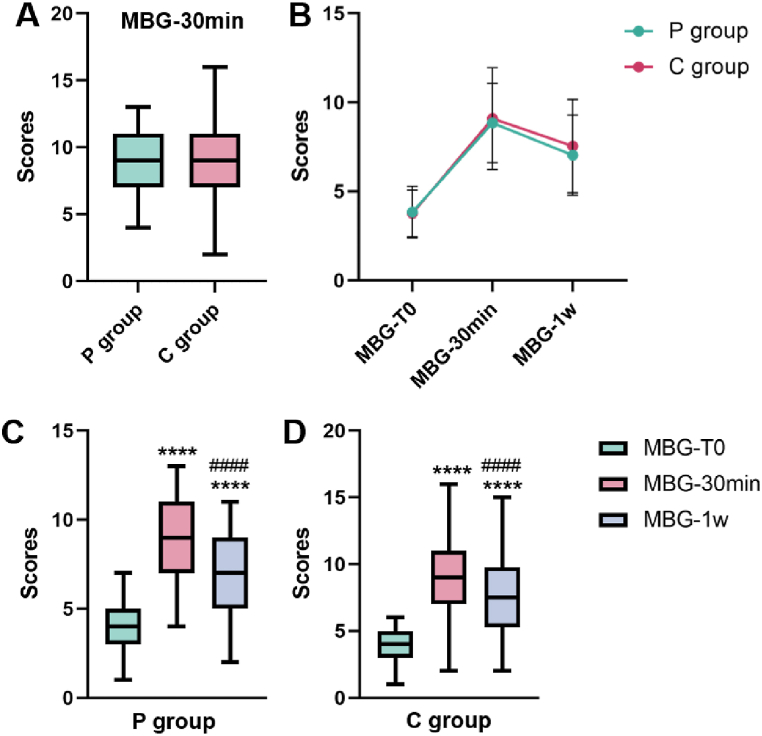
Changes in the MBG score. (A) A box plot of the MBG-30 min score was created for the two groups (*P* = 0.475). (B) A line graph showing the time-dependent changes in the MBG in the two groups. (C) In the P group, compared with those in the MBG-T0, the MBG scores in the MBG-30 min (*P* < 0.0001) and MBG-1w (*P* < 0.0001) significantly increased, while compared with those in the MBG-30 min, the MBG-1w (*P* < 0.0001) significantly decreased. (D) Similarly, in the C group, the MBG score significantly increased at MBG-30 min (*P* < 0.0001) and MBG-1w (*P* < 0.0001) compared to that at MBG-T0, while a significant decrease was observed at MBG-1w compared to that at MBG-30 min (*P* < 0.0001). ****P < 0.0001 versus MBG-T0, ####*P* < 0.0001 versus MBG-30 min. Error bars represent standard deviations.

The MBG-30 min scores for the P group and C group were 9.31 ± 2.27 and 9.53 ± 2.46, respectively. Moreover, there was no statistically significant difference between the two groups (*P* > 0.05, Fig. 2 A). We also found that MBG-30 min and MBG-1w were significantly increased in both groups compared to MBG-T0 (Fig. 2B–D).

### 5.3 Secondary outcomes

One week after sedation, in patients who underwent gastroscopy, the MBG-1w values of the P group and C group were 6.58 ± 1.63 and 7.04 ± 2.49, respectively. The dream rates of patients in both groups were 42.2 % and 40.7 %, respectively, and there was no statistically significant difference between the two groups (*P* > 0.05). Similarly, there were no significant differences in the SAS-1w, PHQ-9-1w, or PQSI-1w scores between the two groups of patients one week after examination (*P* > 0.05; Table 2).

**Table 2:** Dreams, MBGs, and emotions.

	P group (n = 109)	C group (n = 108)	t/χ^2^/Z	*P* value
Dream n (%)	46(42.2)	44(40.7)	0.048	0.827
Dream content			0.186	0.911
Forget	23(50.0)	22(50.0)		
Pleasant	18(39.1)	16(36.4)		
Others	5(10.9)	6(13.6)		
MBG-30min	8.84 ± 2.22	9.09 ± 2.86	−0.715	0.475
MBG-1w	7.04 ± 2.25	7.55 ± 2.63	−1.534	0.126
SAS-1w	39.49 ± 5.43	38.62 ± 6.04	1.111	0.268
PHQ-9-1w	3.65 ± 2.36	3.68 ± 2.18	−0.081	0.936
PSQI-1w	8.24 ± 2.24	7.80 ± 2.08	1.505	0.134

MBG-30 min measurements were obtained 30 min after the endoscopic examination recovery. The SAS-1w, PHQ-9-1w, PSQI-1w, and MBG-1w scores were obtained one week after anesthesia, and the anxiety self-rating scale was used. The values are presented as the means ± SDs or numbers (percentages).

### 5.4 MBG-30 min influencing factors

To further investigate the factors influencing the euphoric reaction, we employed multiple linear regression analysis to explore the potential determinants of the 30 min length of the MBG. After excluding variables with collinearity issues, a linear regression analysis was performed with the MBG-30 min score as the dependent variable and possible influencing factors as independent variables (Table 3). The regression equation yielded significant results, with F = 15.087 and *P* < 0.001. Notably, male sex (*P* = 0.002), AUDIT score (*P* < 0.001), dream status (*P* < 0.001), and examination time (*P* = 0.003) significantly positively predicted MBG-30 min. Collectively, these variables accounted for 49.40 % of the variance in training match satisfaction.

**Table 3:** Multiple linear regression analysis of the MBG-30 min dataset.

	Unstandardized coefficients		Standardized coefficients	t	*P* value.	VIF
	B	SE	β			
Constant	7.304	2.150		3.398	0.001	
Age	−0.014	0.011	−0.062	−1.223	0.223	1.105
Sex(Female)	−0.907	0.286	−0.177	−3.173	0.002	1.326
BMI	0.026	0.044	0.029	0.581	0.562	1.063
Education	0.186	0.169	0.055	1.099	0.273	1.063
Smoking	0.488	0.330	0.079	1.480	0.140	1.228
AUDIT	0.328	0.042	0.442	7.810	0.000	1.366
SAS	−0.009	0.026	−0.025	−0.364	0.716	1.959
PHQ-9	0.070	0.063	0.059	1.104	0.271	1.214
PSQI	−0.048	0.063	−0.038	−0.761	0.447	1.075
Home dream recall frequency	−0.284	0.208	−0.090	−1.362	0.175	1.872
Induction time (s)	0.004	0.017	0.011	0.207	0.836	1.119
Examination time (min)	0.232	0.076	0.166	3.038	0.003	1.280
Awake time(min)	−0.012	0.062	−0.010	−0.202	0.840	1.155
Recovery time(min)	−0.059	0.058	−0.051	−1.024	0.307	1.071
Dream	1.255	0.277	0.242	4.525	0.000	1.226

Male sex, AUDIT score, examination time, and dream status had a significant positive relationship with MBG effects. D-W: 0.924. AdjR^2^ = 0.494. VIF, Variance inflation factor.

### 5.5 Sedative effect

**Fig. 3:**
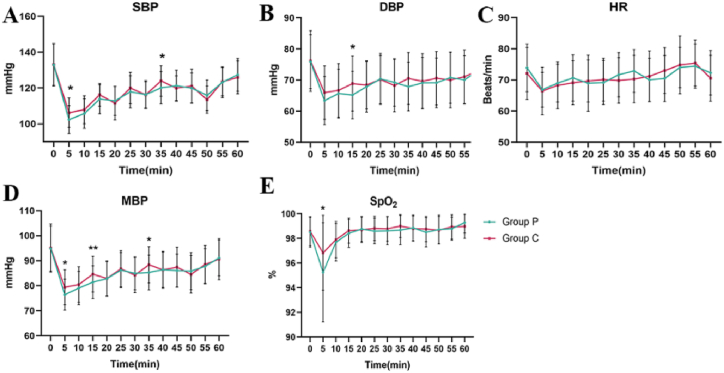
The vital signs of the patients changed dynamically during sedation. (A) Systolic blood pressure was significantly greater in the C group than in the P group at 5 and 35 min post-sedation. (B) Diastolic blood pressure was significantly greater in the C group than in the P group at 15 min post-sedation. (C) At 5, 15, and 35 min post-sedation, the mean arterial pressure in the C group was significantly greater than that in the P group. (D) There was no significant difference in heart rate between the two groups during sedation. (E) SpO_2_ was significantly greater in the C group than in the P group at the beginning of 5 min of sedation. *, the difference between the P group and the C group, *P* < 0.05. **, *P* < 0.01. Error bars represent standard deviations. Error bars represent standard deviations.

We further investigated the differences in sedative effects between propofol and ciprofol and found no statistically significant differences in induction time, examination time, awake time, recovery time, patient satisfaction or endoscopist satisfaction between the two groups (*P* > 0.05; Table 4). However, during sedation, SBP (*P* < 0.001), DBP (*P* = 0.021), MAP (*P* < 0.001), and SpO_2_ (*P* < 0.001) were greater in the C group at T1 (Table 5), while SBP, DBP, MAP, and SpO_2_ were not significantly different between the two groups at T2 (*P* > 0.05). In addition, after hemodynamic monitoring every 5 min during sedation, the hemodynamics in the C group were more stable (Fig. 3A–E).

**Table 4:** Sedative effect.

	P group (n = 109)	C group (n = 108)	t/χ^2^/Z	*P* value
Induction time (s)	57.48 ± 7.69	58.98 ± 7.73	−1.438	0.152
Examination time (min)	8.70 ± 1.71	8.64 ± 1.96	−0.234	0.815
Awake time(min)	8.30 ± 1.75	8.62 ± 2.50	−1.083	0.280
Recovery time(min)	6.50 ± 2.08	7.31 ± 2.28	0.579	0.563
Patient satisfaction	10(8.5,10)	9(8,10)	−0.873	0.383
Endoscopist satisfaction	9(9,10)	9(9,10)	−1.667	0.096

**Table 5:** Hemodynamic measurements.

	Time	P group (n = 109)	C group (n = 108)	t/χ^2^/Z	*P* value
SBP (mmHg)	T0	132.94 ± 11.85	133.08 ± 11.54	−0.093	0.926
	T1	102.40 ± 7.789	106.25 ± 8.242	−3.533	0.001
	T2	122.62 ± 8.88	123.84 ± 9.34	−0.985	0.326
DBP (mmHg)	T0	75.93 ± 8.66	76.16 ± 9.74	−0.185	0.854
	T1	63.43 ± 7.70	66.00 ± 8.60	−2.319	0.021
	T2	70.73 ± 7.88	70.94 ± 8.71	−0.179	0.858
MAP (mmHg)	T0	94.89 ± 9.02	95.11 ± 9.63	−0.175	0.861
	T1	76.45 ± 6.19	79.40 ± 6.99	−3.290	0.001
	T2	87.99 ± 7.01	88.60 ± 7.93	−0.602	0.548
HR	T0	73.88 ± 7.64	72.08 ± 8.41	1.649	0.101
	T1	66.89 ± 5.56	66.45 ± 7.61	0.482	0.630
	T2	72.77 ± 7.04	74.19 ± 8.45	−1.342	0.181
SpO_2_(%)	T0	98.50 ± 1.21	98.58 ± 1.18	−0.487	0.627
	T1	95.25 ± 4.01	96.83 ± 3.04	−3.279	0.001
	T2	98.72 ± 1.16	98.67 ± 1.12	0.375	0.708

SBP, systolic blood pressure; DBP, diastolic blood pressure; MAP, mean arterial pressure; HR, heart rate; SpO_2_, finger pulse oxygen saturation. The data are presented as the mean ± SD.

### 5.6 Adverse drug reactions (ADRs)

There were no significant differences in the incidence of choking cough, involuntary movement, nausea and vomiting, dizziness, bradycardia, or airway intervention during sedation between the C group and the P group (*P* > 0.05). However, there was an increasing trend in the occurrence of hypotension, SpO_2_<95 %, and SpO_2_<90 %, but these differences were not statistically significant. During sedation, the incidence of severe hypotension and injection pain in the C group was significantly lower (*P* < 0.05) (Table 6).

**Table 6:** Adverse drug reactions.

ARDs	P group (n = 109)	C group (n = 108)	t/χ^2^/Z	*P* value
Choking cough n(%)	17(15.6)	11(10.2)	1.413	0.234
Injection pain n(%)	39(35.8)	12(11.1)	18.362	0.000
Involuntary movement n(%)	12(11.0)	18(16.7)	1.457	0.227
Nausea and vomiting n(%)	21(19.3)	17(15.7)	0.467	0.494
Dizziness n (%)	12(11.0)	10(9.3)	0.182	0.669
Bradycardia n(%)	9(8.3)	13(12.0)	0.851	0.356
Hypotension n(%)	44(40.4)	32(29.6)	2.748	0.097
Severe hypotension n(%)	13(11.9)	4(3.7)	5.080	0.024
SpO_2_<95 % n(%)	24(22.0)	14(13.0)	3.080	0.079
SpO_2_<90 % n(%)	15(13.8)	7(6.5)	3.156	0.076
Airway intervention n(%)	7(6.4)	5(4.6)	0.334	0.564

The data are presented as numbers (percentages).

## 6 Discussion

Many clinical studies have shown that nearly half of patients experience euphoric reactions after sedation with propofol during gastrointestinal endoscopy [11,27], which is closely related to addiction [10,13], and that most abusers report pleasure or euphoric reactions when propofol is injected [12,26]. In this prospective randomized controlled trial, we found that patients receiving ciprofol or propofol for sedation during gastroscopy had the same level of euphoria 30 min after awakening and that the level of euphoria associated with both drugs continued to increase for at least 1 week. In addition, there were relatively few adverse reactions to ciprofol, which is consistent with the results of previous studies [17].

These findings are concerning because they suggest that the two drugs may have similar addictive potential. At present, the most serious issue is the high incidence of overdose death caused by propofol in people with drug addictions [15,28], and some scholars have called for the addition of propofol to the list of controlled drugs [16]. On the other hand, we investigated the related factors influencing the euphoria response, such as dreaming, AUDIT score, examination time and male sex. These characteristics can be considered risk factors for addiction, and there is a need to improve people's awareness of these risks and develop strategies to reduce patients' risk of addiction. Considering that ciprofol is increasingly widely used in clinical practice, we must be vigilant about the addictive potential of ciprofol, and further research and additional efforts are needed to reduce the addiction risk of patients and seek adjuvant drugs to reduce the dosage of ciprofol or propofol to ensure the safe and effective use of these drugs in clinical practice. It is believed that with more comprehensive and accurate studies, we can better understand the euphoric reactions of patients during sedation in patients undergoing gastroscopy and provide more effective sedation strategies.

Alternatively, the negative results of the present study may be related to the mechanism of action of these two drugs. Propofol is an intravenous general anesthetic drug that has sedative effects by increasing the inhibitory effect of GABA [29], while ciprofol is a short-acting intravenous sedative based on the structural modification of propofol, which may have the same mechanism of action as propofol [21]. Moreover, the specific mechanism is unclear. Scholars believe that the euphoric reaction and its trigger are associated with increased activity in the dopamine reward system [30]; therefore, future research could further explore the pharmacological properties of these two drugs to determine the underlying mechanism involved [29,31].

The present study has several limitations. First, the results of this study may be related to the sample size. We used a single-center randomized controlled trial design, and the sample of this study was obtained from a single medical center, which may have certain limitations. Because of differences in clinical practice and patient characteristics across centers, the results may not be generalizable to other centers or to different patient populations. Future studies could consider multicenter collaboration to increase the diversity and representation of the sample. Second, we studied only two drugs, propofol and ciprofol, and did not include other drugs that may have an effect on the euphoric reaction. Therefore, our conclusions apply only to gastroscopy patients sedated with propofol and ciprofol. Third, intravenous bolus drug administration was used in this study, and previous studies have shown a correlation between the pump rate and patients' euphoric reactions [10]; however, the results of the present investigation were not able to confirm these findings. This may be because the rate of bolus propofol can be adjusted more flexibly according to the specific condition of the patient, while the rate of pump propofol is relatively fixed and difficult to adjust according to individual patient differences. Intravenous injection of drugs can improve patient compliance through individual differences. Whether these two methods of administration lead to different euphoric reactions after examination is worthy of further exploration. In addition, the depth of anesthesia during sedation also needs to be examined. We considered the cost effectiveness of BIS monitoring during gastroscopy. BIS monitoring of sedation levels was not performed. Patients whose depth of sedation affects their euphoric reaction should be further explored. Finally, we did not investigate the duration of the euphoric reaction in the two groups after the examination, and subsequent studies are worth exploring further.

## 7 Conclusion

This prospective randomized, double-blind, controlled trial revealed that patients receiving ciprofol or propofol for sedation during gastroscopy had the same euphoric reaction (with male sex, examination time, AUDIT score, and dreaming as risk factors for euphoric reaction) and that there were significantly fewer ciprofol-induced adverse reactions.

## Funding information

This study was supported by grants from the 10.13039/100017962Jiangsu Commission of Health (JSDW202231).

## Ethical approval statement

The study protocol was approved by the Ethics Committee of Xuzhou Renci Hospital (XZRCLL-KT-202307001) and strictly adhered to the ethical principles outlined in the Declaration of Helsinki.

## Informed consent

All patients who participated in the clinical trial provided written informed consent.

## Data availability statement

All data generated or analyzed during this study are included in this article, and you can contact the corresponding author with any additional questions.

## CRediT authorship contribution statement

**Teng Li:** Writing – original draft, Software, Methodology. **Jin Zhang:** Writing – review & editing, Visualization. **Zhouliang Liu:** Formal analysis, Data curation. **Yao Lu:** Methodology, Formal analysis, Data curation. **Chuhao Gong:** Formal analysis, Data curation. **Dan Han:** Supervision, Software, Methodology. **Ying Wu:** Methodology, Data curation. **Kailun Gao:** Investigation, Formal analysis, Data curation. **Lei Heng:** Visualization, Validation, Supervision, Project administration, Investigation, Conceptualization. **Liwei Wang:** Writing – review & editing, Funding acquisition, Conceptualization. **Peng Peng:** Writing – review & editing, Visualization, Validation, Supervision, Resources, Project administration, Methodology, Investigation, Conceptualization.

## Declaration of competing interest

The authors declare that they have no known competing financial interests or personal relationships that could have appeared to influence the work reported in this paper.
