# Supplementary material for: Effect of propofol and ciprofol on the euphoric reaction in patients with painless gastroscopy: A prospective randomized controlled trial
Source: Heliyon. 2024 Apr 25;10(9):e30378. doi: 10.1016/j.heliyon.2024.e30378 (PMC11068811; doi:10.1016/j.heliyon.2024.e30378)
Supplement: Multimedia component 1 [file mmc1.pdf]

Form to confirm authorship changes for *Heliyon*

This form must be ***signed by all authors*** when there is a change in authorship which includes changes to any of the following items: author name(s), order of the authors, the corresponding author(s), the addition of authors, the removal of authors and changes in affiliation.

By personally signing this note, **all** authors confirm that: I) the changes are in accordance with their scientific contribution, II) they agree with all the changes and III) confirm that the authorship list conforms to the authorship criteria outlined on*Heliyon*'s ethics page. IV) it is the responsibility of the corresponding author to get the signature from all co-authors accepting the change. In case of any ethic violation/malpractice in the signature, the corresponding author is accountable. The completed form should be returned along with the final/revised manuscript to proceed further with the manuscript. Manuscripts for which incomplete forms have been submitted will be rejected within 5 working days.

Please include the author's name in the correct order, followed by author's who should be removed from the manuscript.

Any disputes on the authorship list and contributions need to be resolved by the involved scientists and *Heliyon* will only proceed with the evaluation of the manuscript once we receive confirmation, through this form, that such an agreement between the authors has been reached.

***Heliyon* will not accept changes to the authorship list in the late stages of the editorial process (when a paper is in Accept in Principle stage, acceptance or after publication)**

Manuscript number: HELIYON-D-23-63019R2

Article title:Effect of Propofol and Ciprofol on the Euphoric Reaction in Patients with Painless Gastroscopy: A Prospective Randomized Controlled Trial

Complete new author list:Teng Li, Jin Zhang, Zhouliang Liu, Yao Lu, Chuhao Gong, Dan Han, Ying Wu, Kailun Gao, Lei Heng, Liwei Wang, Peng Peng

Date:2024/04/23

#	First name	Last name	Dept. & Institution name	Institutional email address	Order change (Y/N)	Addition / Deletion	Change in Author name (Y/N)	Affiliation Change (Y/N)	Reason for the change	Signature
1	Teng	Li	XuZhou Clinical School of Xuzhou Medical University, Xuzhou, China	light0810@163.com	N		N	N		Li Teng

2	Jin	Zhang	XuZhou Clinical School of Xuzhou Medical University, Xuzhou, China	301106120825@stu.xzhmu.edu.cn	Y	Addition			The authorship change is prompted by the substantial assistance provided by Jin Zhang during the initial revision process. Her contributions have been invaluable, encompassing critical insights, data analysis, significant revisions, and extensive literature review, all of which have greatly enhanced the quality and clarity of the manuscript.	Zhang. Jin
3	Zhouliang	Liu	XuZhou Clinical School of Xuzhou Medical University, Xuzhou, China	1181650840@qq.com	N		N	N		Liu zhouliang
4	Yao	Lu	XuZhou Clinical School of Xuzhou Medical University, Xuzhou, China	1336492763@qq.com	N		N	N	Following substantial revisions to the manuscript post-initial review, the authorship sequence was adjusted accordingly to accurately acknowledge each author's significant contributions based on their respective involvement in the revision process.	Lu Yao
5	Chuhao	Gong	Department of Anesthesiology, Xuzhou Renci Hospital, Xuzhou, China	240034117@qq.com	N		N	N		Gong Chuhao

6	Dan	Han	Department of Anesthesiology, Xuzhou Renci Hospital, Xuzhou, China	handants@sina.com	N		N	N		Han Dan
7	Ying	Wu	XuZhou Clinical School of Xuzhou Medical University, Xuzhou, China	wuyingdoctor@163.com	N		N	N		Wu Ying
8	Kailun	Gao	XuZhou Clinical School of Xuzhou Medical University, Xuzhou, China	592551952@qq.com	N		N	N		Gao Kailun
9	Lei	Heng	Department of Anesthesiology, Xuzhou Cancer Hospital, Xuzhou, China	258519729@qq.com	N		N	N		Heng Lei
10	Liwei	Wang	XuZhou Clinical School of Xuzhou Medical University, Xuzhou, China.<br><br>Department of Anesthesiology, Xuzhou Central Hospital, Xuzhou, China	doctorlww@sina.com	Y		N	N	This is due to our mistake, which mistakenly set Liwei Wang as the corresponding author, while Peng Peng contributed relatively much, and all the authors agreed to change the corresponding author	Wang, LiWei
11	Peng	Peng	Department of Anesthesiology, Xuzhou Renci Hospital, Xuzhou, China	240034117@qq.com	Y		N	N	This is due to our mistake, which mistakenly set Liwei Wang as the corresponding author, while Peng Peng contributed relatively much, and all the authors agreed to change the corresponding author.	Peng Peng
